# Supplementary material for: MetaRibo-Seq measures translation in microbiomes
Source: Nat Commun. 2020 Jun 29;11:3268. doi: 10.1038/s41467-020-17081-z (PMC7324362; doi:10.1038/s41467-020-17081-z)
Supplement: Supplementary file 10 — Supplementary Data 7 [file 41467_2020_17081_MOESM10_ESM.zip › File2/Confidence_VeryHigh_Taxonomy/288943_out.krona.html]

Javascript must be enabled to view this page.

members
magnitude
magnitudeUnassigned
count
unassigned
taxon
rank

288943\_out

15

15
superkingdom
2

1239
phylum
15

186801
class
15

15
186802
order

1897047
species

SRS147271\_contig\_number\_21145
1

13
541000
family

13

SRS098717\_contig\_number\_contig-100\_3673.112332
1
genus
946234

9

SRS014979\_contig\_number\_contig-100\_20720.65428SRS016132\_contig\_number\_5489SRS075984\_contig\_number\_contig-100\_24885.64420SRS097889\_contig\_number\_35319SRS1041091\_contig\_number\_13720SRS1041136\_contig\_number\_26713SRS1041138\_contig\_number\_14491SRS143466\_contig\_number\_27924SRS143780\_contig\_number\_23999
1193534
species

292800
species

SRS013098\_contig\_number\_36424SRS147766\_contig\_number\_12111SRS148511\_contig\_number\_17887
3

1
family
186803

1
658087
species

SRS104197\_contig\_number\_106
